# Optimization of Trash Identification on the House Compound Using a Convolutional Neural Network (CNN) and Sensor System

**DOI:** 10.3390/s23031499

**Published:** 2023-01-29

**Authors:** Emil Naf’an, Riza Sulaiman, Nazlena Mohamad Ali

**Affiliations:** 1 Institute of IR4.0, Universiti Kebangsaan Malaysia, Bangi 43600, Malaysia; 2 Faculty of Computer Science, Universitas Putra Indonesia YPTK Padang, Padang 25221, Indonesia

**Keywords:** optimization, identification, trash, Convolutional Neural Network (CNN), sensor

## Abstract

This study aims to optimize the object identification process, especially identifying trash in the house compound. Most object identification methods cannot distinguish whether the object is a real image (3D) or a photographic image on paper (2D). This is a problem if the detected object is moved from one place to another. If the object is 2D, the robot gripper only clamps empty objects. In this study, the Sequential_Camera_LiDAR (SCL) method is proposed. This method combines a Convolutional Neural Network (CNN) with LiDAR (Light Detection and Ranging), with an accuracy of ±2 mm. After testing 11 types of trash on four CNN architectures (AlexNet, VGG16, GoogleNet, and ResNet18), the accuracy results are 80.5%, 95.6%, 98.3%, and 97.5%. This result is perfect for object identification. However, it needs to be optimized using a LiDAR sensor to determine the object in 3D or 2D. Trash will be ignored if the fast scanning process with the LiDAR sensor detects non-real (2D) trash. If Real (3D), the trash object will be scanned in detail to determine the robot gripper position in lifting the trash object. The time efficiency generated by fast scanning is between 13.33% to 59.26% depending on the object’s size. The larger the object, the greater the time efficiency. In conclusion, optimization using the combination of a CNN and a LiDAR sensor can identify trash objects correctly and determine whether the object is real (3D) or not (2D), so a decision may be made to move the trash object from the detection location.

## 1. Introduction

A clean house compound is a dream for everyone. Trash can be caused by leaves falling from trees or plastic trash such as plastic bottles and snack packets. Usually, trash on the house compound is cleaned by the owner or the person assigned. If homeowners are busy and do not have time to clean the house compound, then the house will look unclean because trash is scattered in the house compound.

Currently, the state considers essential urban services, such as water, sanitation, and solid waste management, to be the responsibility of local or national governments [1,2,3,4]. Research on the identification and classification of trash has been performed [5,6,7]. However, it was not optimal regarding the amount of trash detected and accurate trash detection. At the same time, optimization plays an essential role in computer vision because many computer vision algorithms employ an optimization step at some point in their proceeding [8]. A study was conducted by Fuchikawa in 2005 on trash collection using an OSR robot (Outdoor Service Robot) [9]. Unfortunately, this research was only aimed at collecting plastic trash in PET bottles. Another study was conducted by an Italian research group led by Mazzolai [10]. They named the robot DustCart. This robot groups junk types based on user input. After the user inputs the type of trash, the robot opens the trashed store according to the type of trash input. However, robot cameras are for navigation, avoiding obstacles, and not classifying trash types.

Improvements in the reliability and processing speed of the vision system and experiments with other trash must be conducted [9]. Improvement was achieved at Stanford University through a study on trash classified using the CNN (Convolution Neural Network) method [11]. However, the amount of trash that can be identified is only up to six items of trash [12,13,14], while there are more than six items in the house compound.

**Figure 1:**
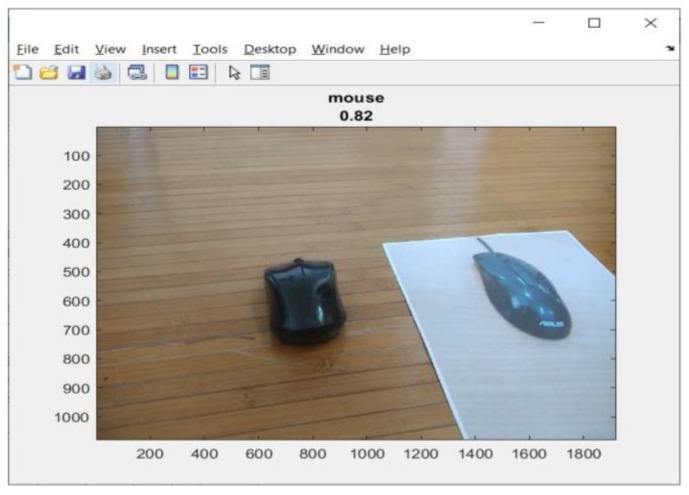
A sample detection object (mouse) in 2D and 3D.

Identification of trash is an essential step before separation, and this can be performed efficiently with the help of different machine-learning and image processing algorithms. A Convolutional Neural Network (CNN) is preferred for the classification of images [15]. However, most methods of identifying objects cannot distinguish whether the object is a real (3D) image or an image in the form of a photograph on paper (2D) [12,16,17,18,19,20]. Figure 1 shows a test object (mouse) in 2D and 3D. The object is detected using a Convolutional Neural Network (CNN). The test results show that 2D and 3D (real) objects can be detected well, with a prediction accuracy of 82%. In Figure 1, the 2D object is located on the right and the 3D object is located on the left.

**Figure 2:**
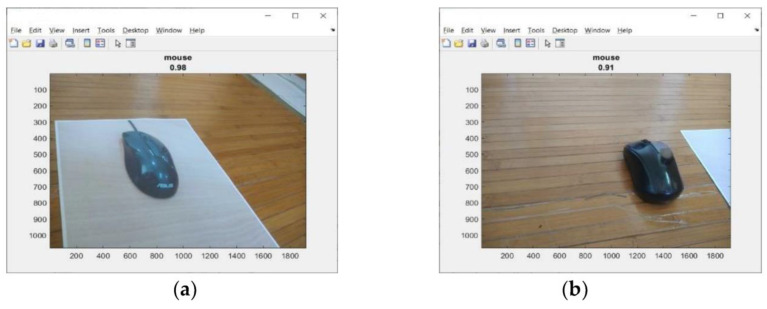
A sample of the detection object (mouse). (**a**) A photo of a mouse on paper (2D); (**b**) a real mouse photo (3D).

Then, each object is detected, 2D objects and 3D objects, using the Convolutional Neural Network (CNN). An Intel Core i7 (8th gen) laptop, with 16 GB RAM, a NVIDIA GeForce RTX2060 graphics card and MATLAB 2020a software was used. For 2D objects, the samples are photos of objects on paper. The test results for 2D objects can be seen in Figure 2a. The test results were obtained with a prediction accuracy of 98%.

Furthermore, testing was carried out for 3D objects (real). This object was placed next to the 2D object. The test results can be seen in Figure 2b. The test results were obtained with a prediction accuracy of 91%. From these three test results, it can be said that the Convolutional Neural Network (CNN) can detect objects well. This will be a problem if the object is moved from one place to another using the robot gripper. How would it be if the robot wanted to move the trash object while the trash object was just a photograph of trash on paper? A robot must identify/recognize real (3D) trash objects.

In any application, we can use a particular classifier and try to optimize its performance. The usual approach is to try several different classifiers and choose the one that performs the best on a separate validation set [21]. In this case, we propose a Convolutional Neural Network (CNN) combined with LiDAR sensors to identify whether the object is real (3D) or not (2D). A CNN was chosen because, at present, a CNN is the best machine-learning method in object identification. Therefore, this study proposes optimizing trash identification in the house compound using a Convolutional Neural Network (CNN).

The proposed research has similarities with sensor fusion. In sensor fusion, several sensors are combined to take measurements. The goal is that the resulting data are more accurate than using only one sensor. In this study, a camera and a LiDAR sensor were used. Many studies use a combination of LiDAR sensors and cameras. Table 1 shows a comparison of their use [22].

## 2. Materials and Methods

**Figure 3:**
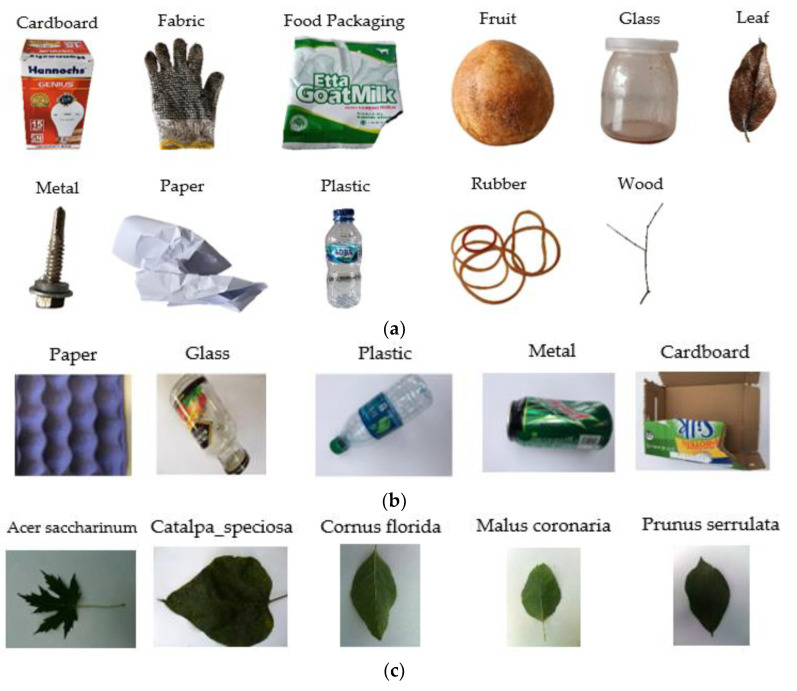
(**a**) Sample images of own trash dataset. (**b**) Sample images of the TrashNet dataset. (**c**) Sample images of the LeafSnap dataset.

In this study, data collection is performed using own trash dataset, the TrashNet dataset [11], and the Leafsnap dataset [32]. These data are grouped into 11 types of data, namely, cardboard, fabric, food packaging, fruit, glass, leaf, metal, paper, plastic, rubber, and wood. Figure 3 shows sample images of the trash dataset. The TrashNet dataset was created by Gary Thung and Mindy Yang. This is a small dataset and contains 1989 images. The LeafSnap dataset was created by Kumar et al. [33]. The dataset consists of 7500 field-augmented images [34]. Own trash datasets were captured by a mobile device (iPhone). The image data are augmented by a laptop using MATLAB R2020a software. This own dataset is used to complement the TrashNet dataset and LeafSnap dataset to obtain 11 types of data.

The data were tested with four pre-trained Convolutional Neural Network (CNN) architectures: AlexNet, VGG16, GoogleNet, and ResNet18. The training parameters can be seen in Table 2.

**Figure 4:**
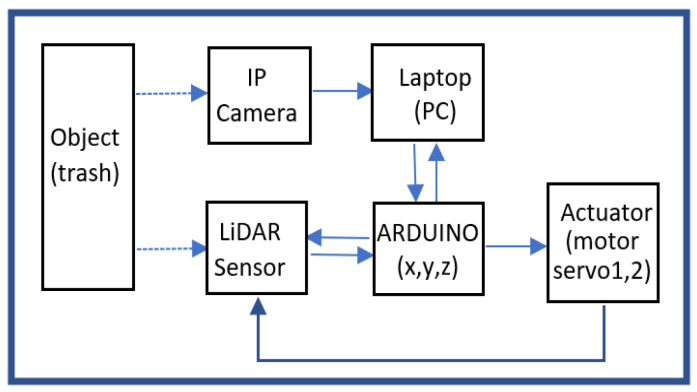
Block diagram.

The training parameters used are those with standard values already existing in the pre-trained CNN architecture. After the data are collected, the next step is to capture the trash object. Figure 4 depicts the block diagram of the proposed system.

**Figure 5:**
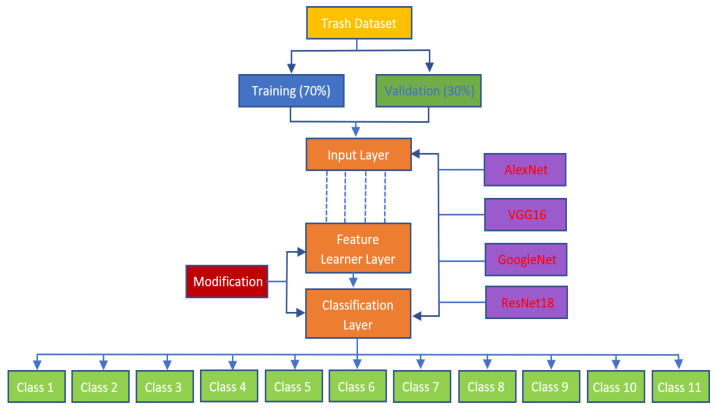
Simple transfer learning using CNN architecture (AlexNet, VGGG16, GooogleNet, and ResNet18).

After the IP camera captures the image data, the MATLAB program resizes the image. It is helpful for further processing on CNN Image Detection. The CNN architecture used can classify up to 1000 types of objects. However, modifications are made to the feature learner to speed up classifying trash objects. The original value is 1000 fully connected layers changed to 11 fully connected layers. This value is changed on every architecture—AlexNet, VGG16, GoogleNet, and ResNet18. Figure 5 depicts transfer learning using the CNN architecture. In the Figure, it can be seen that the first step of the trash dataset is loading.

Furthermore, this dataset is divided into training and validation datasets with a 70:30 ratio. Then, this dataset image is resized according to the size of the input layer architecture used. For AlexNet, the image size is 227 × 227. For VGG16 GoogleNet and ResNet18, the image size is 224 × 224, respectively. The next step is to modify the feature learner layer and classification layer. Fully connected, it is changed from 1000 to 11, so the output from the classification layer will automatically be 11 classes.

**Figure 6:**
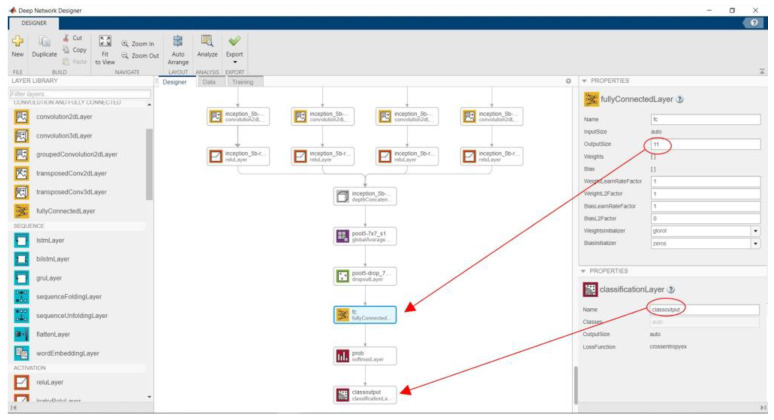
Modifying the GoogleNet architecture using Deep Network Designer.

In MATLAB, the modification process can be performed by changing the architectural design using Deep Network Designer. Figure 6 shows the process of modifying the GoogleNet architecture. 

**Figure 7:**
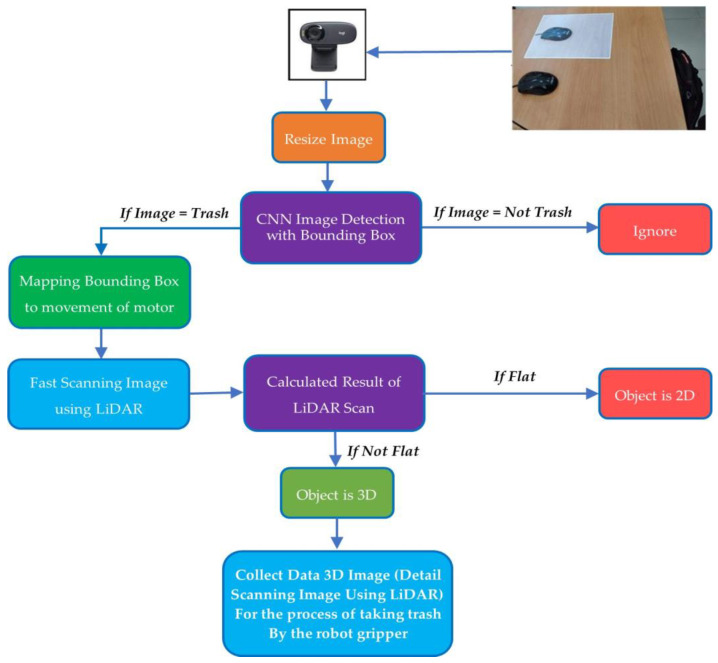
Flowchart of the proposed study.

Furthermore, the MATLAB program ensures that an image is a trash object. If the object is not trash, it is ignored. If it is trash, the laptop (PC) sends a command to Arduino to activate the actuator (servo1 and servo2). This command is based on the detected object bounding box value. The actuator moves the LiDAR sensor in *X* and *Z* coordinates based on the value. After that, the data from the LiDAR sensor are sent by Arduino to the laptop (PC). The proposed flowchart can be seen in Figure 7.

**Figure 8:**
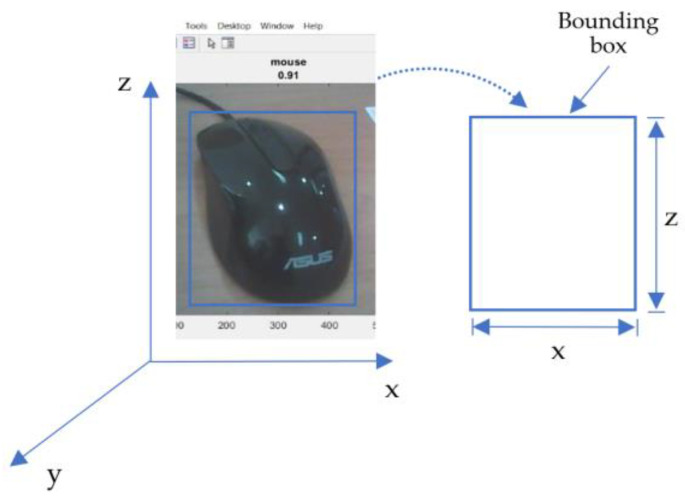
Convert the bounding box to *x*, *y*, and *z* coordinates.

The Mapping Bounding box is used to convert the movement of the servo motor to match the object’s size to be detected, as shown in Figure 8 [35]. After obtaining the *x* and *z*, it is calculated/mapped to the degree of motor servo movement. In the scanning process, servo motor 1 (*x*) and servo motor 2 (*z*) move at the boundary in the bounding box.

In the designed system, the LiDAR sensor is mounted on top of a servo motor 1. The scanning process is then carried out using a LiDAR sensor. The scanning process is carried out in 2 stages, namely, the fast and detailed scanning processes. This is to achieve an efficient scanning time. If the detected object is flat, then the object is a 2D object. If it is 2D, the detailed scanning process does not need to be performed. However, if the object is not flat, the detected object is a 3D object. If the object is 3D, a detailed process needs to be performed. It aims to determine with certainty the geometric shape of the object. With the object’s geometry data, the robot gripper can easily lift trash objects.

### 2.1. TF40 LiDAR

**Figure 9:**
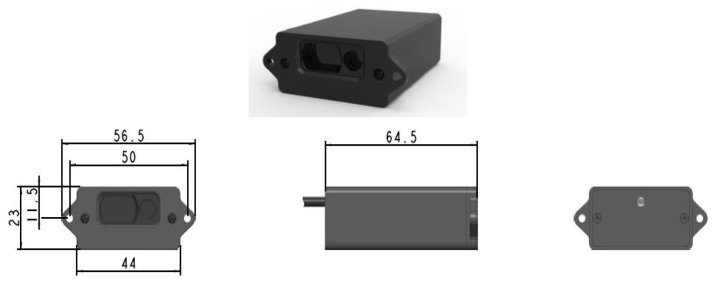
TF40 LiDAR.

TF40 is an millimeter-level accuracy LiDAR with a range of up to 40 m [36,37,38]. Accuracy in mm is essential because the trash object is relatively small, 15 cm × 20 cm. It is adjusted to the width of the robot gripper. TF40 has the following features: high accuracy, tiny, small light spot, visible laser, and easier aiming. Table 3 is the main parameter of TF40, and Figure 9 shows the physical form of the TF40 LiDAR sensor and its dimensions.

### 2.2. Fast Scanning Image Using LiDAR

**Figure 10:**
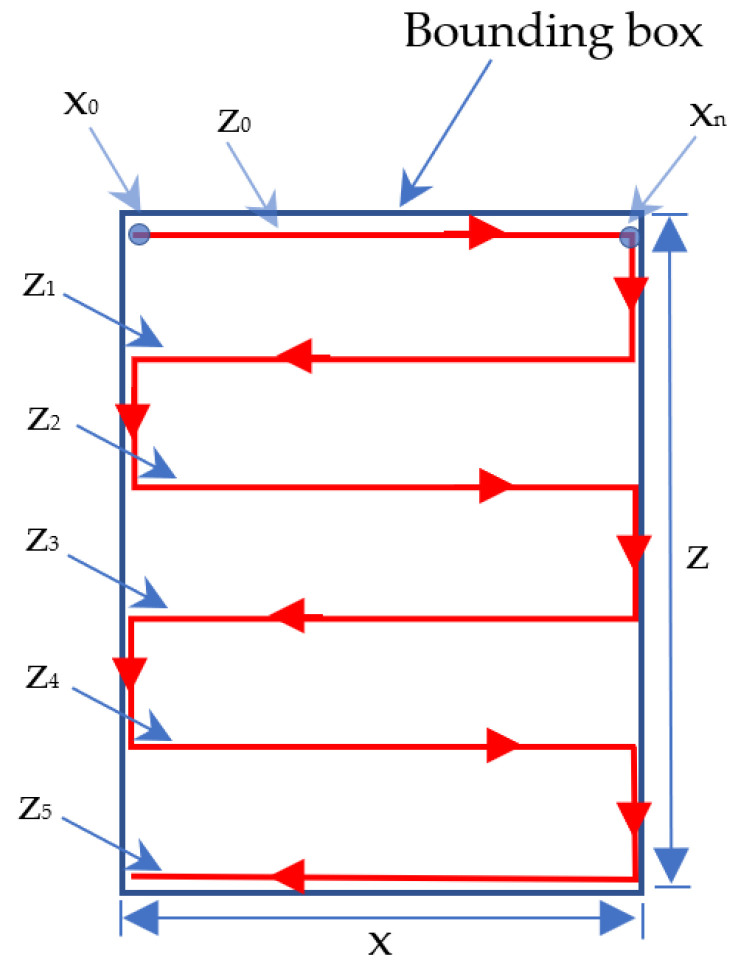
Fast scanning image process path.

The scanning process is fast, from the top left to the bottom right. The fast scanning process is carried out five times according to the path in Figure 10.

The height of the bounding box (*z*) can be different depending on the size of the detected trash object. However, the value of the degree of movement of the servo motor 2 (*z*) in this fast scanning system is obtained from the height of the bounding box divided by five, as shown in the following formula:

(1) $$\mathrm{Servo}\;\mathrm{motor}\;2\;\left(z\right)\mathrm{degree}\;=\frac{\mathrm{Height}\;\mathrm{of}\;\mathrm{bounding}\;\mathrm{box}}{5}$$

After obtaining the value of *x* and *z* based on the bounding box, it is calculated (mapped) to the degree of motor servo movement. Point *x*_0_ line *z*_0_ in Figure 10 is the starting point of the fast scanning image process. If the position of the servo motor 1 and servo motor 2 is not at that point, then the program contained in the Arduino will move them to that position.

**Figure 11:**
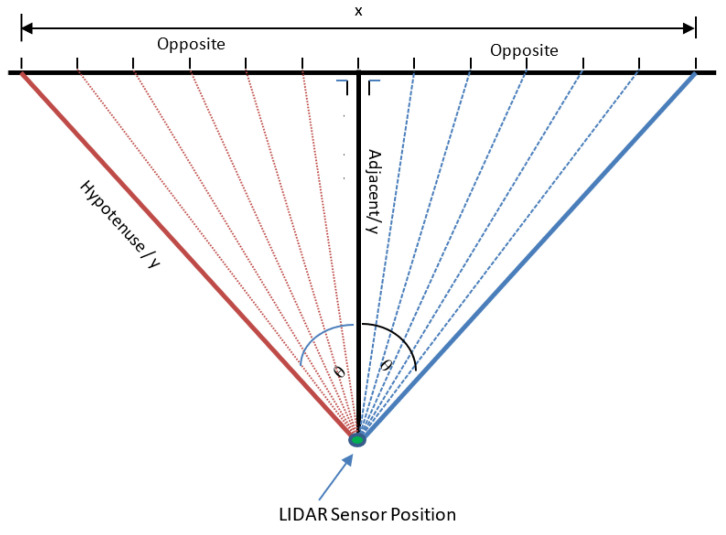
The position of the LiDAR sensor during the scanning process.

After that, servo motor 1 (*x*) and servo motor 2 (*z*) move, as shown in Figure 11 in line z_0_. In the initial conditions, the servo motor will move from point *x*_0_ line *z*_0_ to point xn line z_0_. Then, the servo motor will move down along the servo motor 2 (*z*) degrees. For example, if the height of z is 100 degrees, then the servo motor 2 (*z*) will move down 20 degrees (100 degrees/5 = 20 degrees). The next step is servo motor 1 (*x*) will move along *z*_1_ in the direction of movement opposite to line *z*_0_. The movement of the servo motor is continued by following lines *z*_1_, *z*_2_, *z*_3_, and *z*_4_ and arriving at line *z*_5_ at point *x*_0_.

In Figure 11, the position and movement of the LiDAR sensor during the scanning process are shown. The following algorithm determines whether the detected object has flat, concave, or convex sides.

- If sensor_value = hypotenuse, then the line/point of the image is a flat plane;
- If sensor_value > hypotenuse, then the lines/dots of the image become concave;
- If sensor_value < hypotenuse, then the lines/dots of the image become convex.

**Figure 12:**
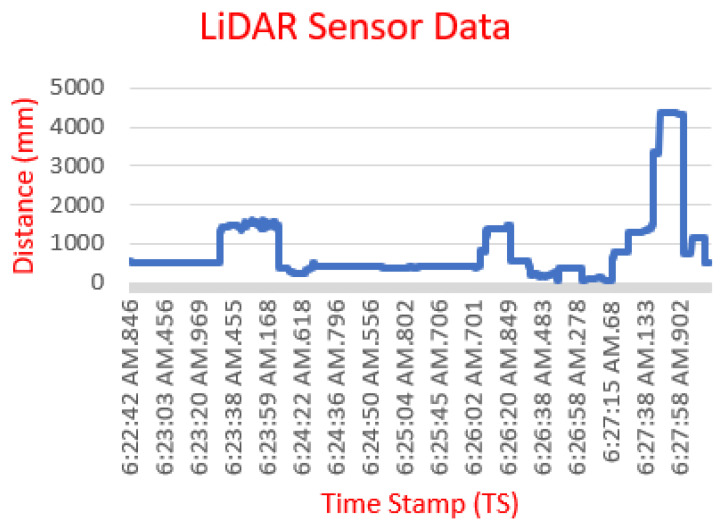
Example of LiDAR sensor data.

In the fast scanning process, the data read is time data (Time Stamp) and distance data (Distance). Figure 12 is an example of a graph of the LiDAR TF40 sensor reading.

**Figure 13:**
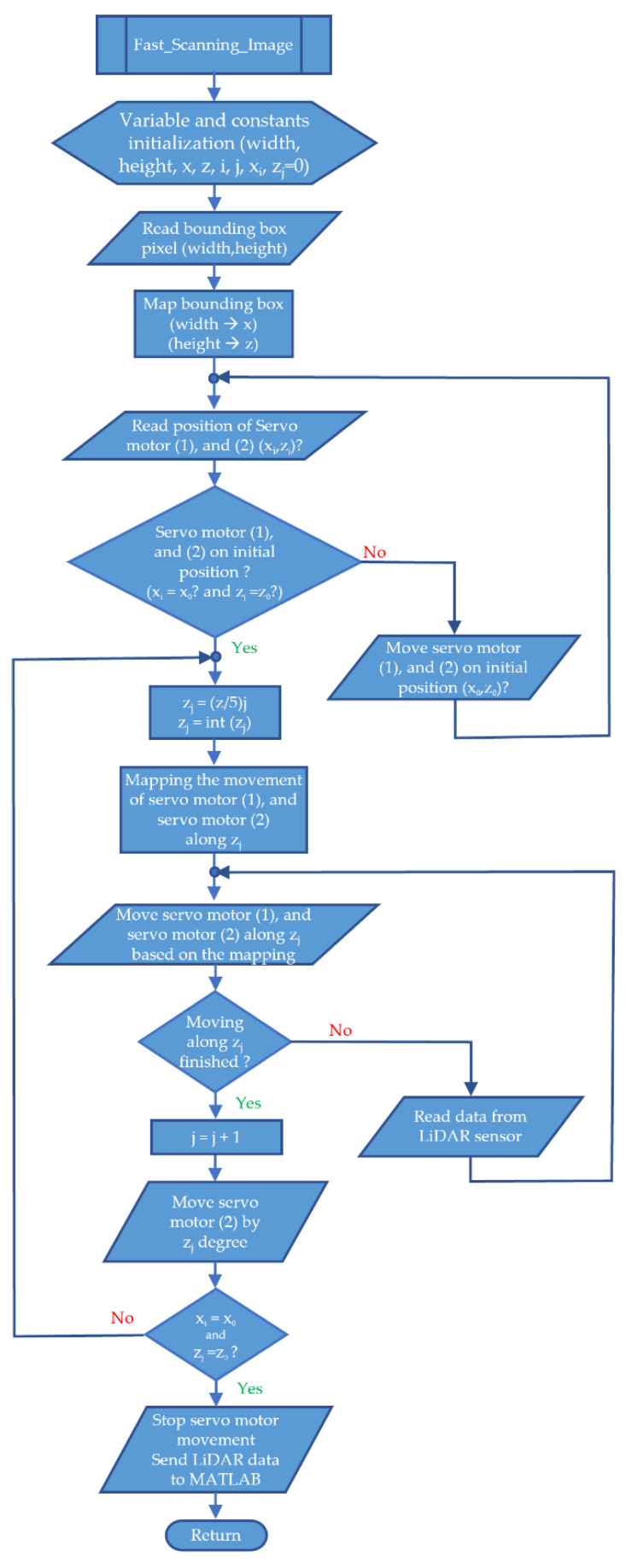
Flowchart of fast scanning image.

Figure 13 is a flowchart of a fast scanning image. This flowchart is in the form of a procedure that is called after the MATLAB program detects the trash object. 

This fast scanning image flowchart is used on Arduino. In the early stages, initialization of the variables and constants that will be used is carried out. Next, the program will read the value of the bounding box pixel (width, height). After this value is known, bounding box mapping is performed. The results of this mapping are in the form of *x* and *z* values, which are the basic values for the degree of movement of the servo motor 1 and servo motor 2. The next step is to read the servo motor’s position (1) and servo motor 2. If the position is not yet in the initial position, then the servo motor 1 and servo motor 2 will be driven in that initial position. Then, the *z_j_* value is divided by 5. If the *z_j_* value is a fractional number, then this value will be rounded off with the int(*z_j_*) command. The servo motor can only move in integer degrees (not floating point degrees). The next step is to map the movement of the servo motor 1 and servo motor 2.

The servo motor uses this mapping to move from point *x*_0_ line *z*_0_ to point *x*_0_ line *z*_5_. Simultaneously with the movement of the servo motor along the *z*_0_, *z*_1_, *z*_2_, *z*_3_, *z*_4_, and *z*_5_ lines, LiDAR sensor data are also read and stored in Arduino memory. After the servo motor 1 and servo motor 2 arrive at point *x*_0_ line *z*_5_, the LiDAR sensor data are sent to the MATLAB program. In MATLAB, the LiDAR sensor data are processed. The result can be 2D or 3D. By the flowchart of the proposed study in Figure 7, if it is 2D, it is ignored. If it is in 3D, then the next step is to do a detailed scanning image process.

### 2.3. Detail Scanning Image Using LiDAR

**Figure 14:**
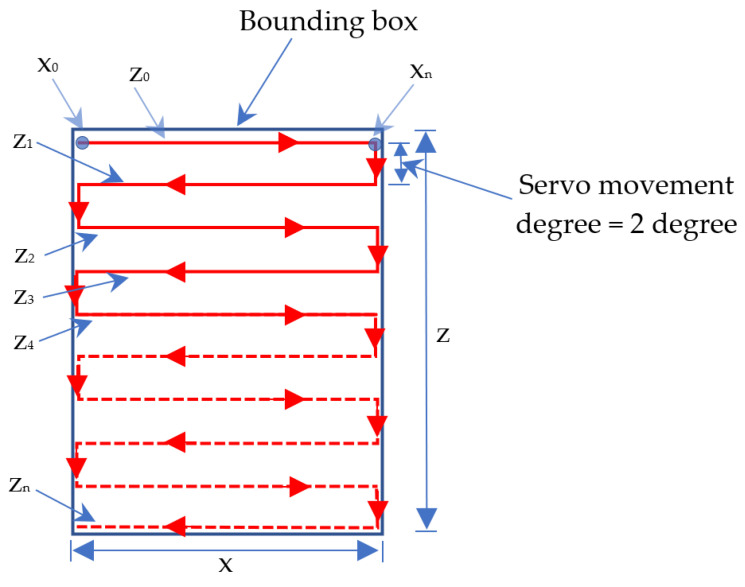
Detail scanning image process path.

Detail scanning is helpful for the process of taking objects using a robot gripper. If the gripper is not positioned correctly, the lifting process may fail. The scanning process is almost the same as fast scanning, but the degree of movement of the servo motor has been determined from the start, which is 2 degrees. The details can be seen in Figure 14.

The difference in the process path between a fast scanning image and a detailed scanning image lies only in the number of lines (*z*). In fast scanning images, the number of lines is limited to 5 lines. Meanwhile, the minimum number of lines for detailed scanning images is five. If the spacing between rows is limited to 2 degrees, then the degree of movement of the servo motor 2 is at least 10 degrees.

**Figure 15:**
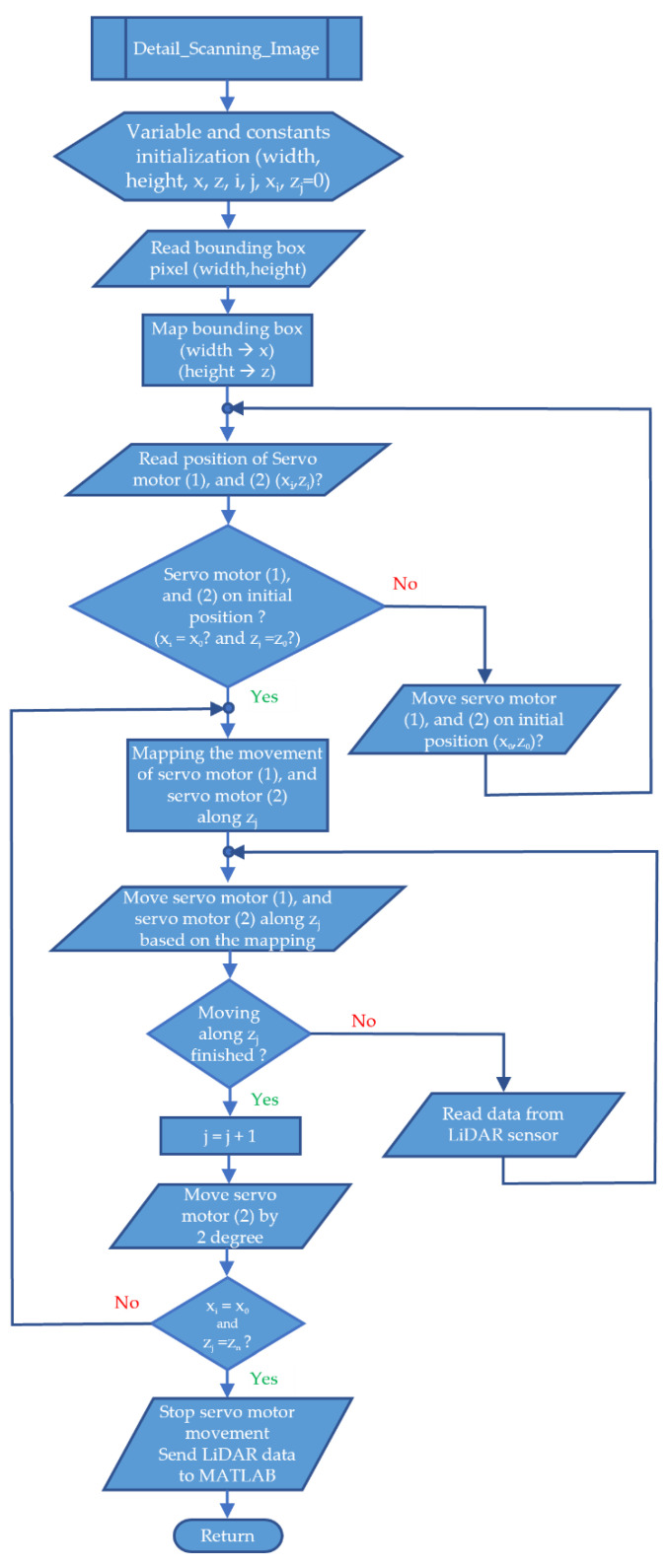
Flowchart of detail scanning image.

Figure 15 is a flowchart of a detail scanning image. This flowchart is in the form of a procedure that is called after the MATLAB program detects the trash object. This flowchart is similar to the flowchart in Figure 13. The difference is that the scanning process will stop if *x_i_* = *x*_0,_ and *z_j_* = *z_n_*. Another difference is that the degree of movement of the servo motor 2 in fast scanning is determined by the z value divided by 5. Meanwhile, in the detail scanning image, the degree of movement is equal to 2 degrees.

In this study, four parameters were used to evaluate the accuracy of the model, namely accuracy (Ac), precision (Pr), recall (Re), F1 score (F1) [39]. The formula can be seen in the following equation.

(2) $$\mathrm{Accuracy}\;=\frac{TP+TN}{TP+FP+TN+FN}$$

(3) $$\mathrm{Precision}\;=\frac{TP}{TP+FP}$$

(4) $$\mathrm{Recall}\;=\frac{TP}{TP+FN}$$

(5) $$F1\;\mathrm{Score}\;=\frac{2\times precision\times recall}{precision+recall}$$

where *TP* is short for the number of true positives. *TN* is short for the number of true negatives. False positive is defined as *FP*. *FN* presents false negatives.

## 3. Results

Image data were tested for 11 types of trash using four CNN architectures, namely AlexNet, VGG16, GoogleNet, and ResNet18. The number of images for each type of trash is 150 pieces. The data are augmented into 1200 images, so the total image is 13,200. The data are divided into 70% training data and 30% test data.

**Figure 16:**
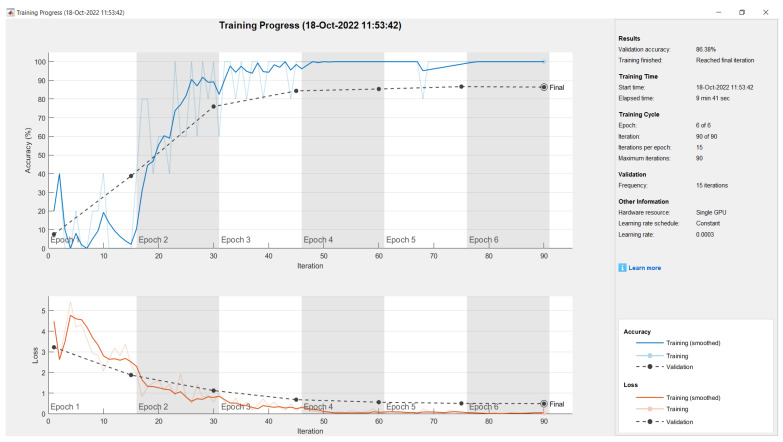
Result of training progress trash object using GoogleNet.

Figure 16 is the result of training progress for GoogleNet. The validation accuracy values for each CNN architecture can be seen in Table 4.

Table 4 shows that the CNN GoogleNet architecture has the highest validation accuracy value, while the CNN AlexNet architecture has the lowest validation value. However, in terms of training time, the fastest training time was achieved by AlexNet and the slowest training time was the VGG16 architecture. 

### 3.1. Confusion Matrix for Trash Classification Testing

**Figure 17:**
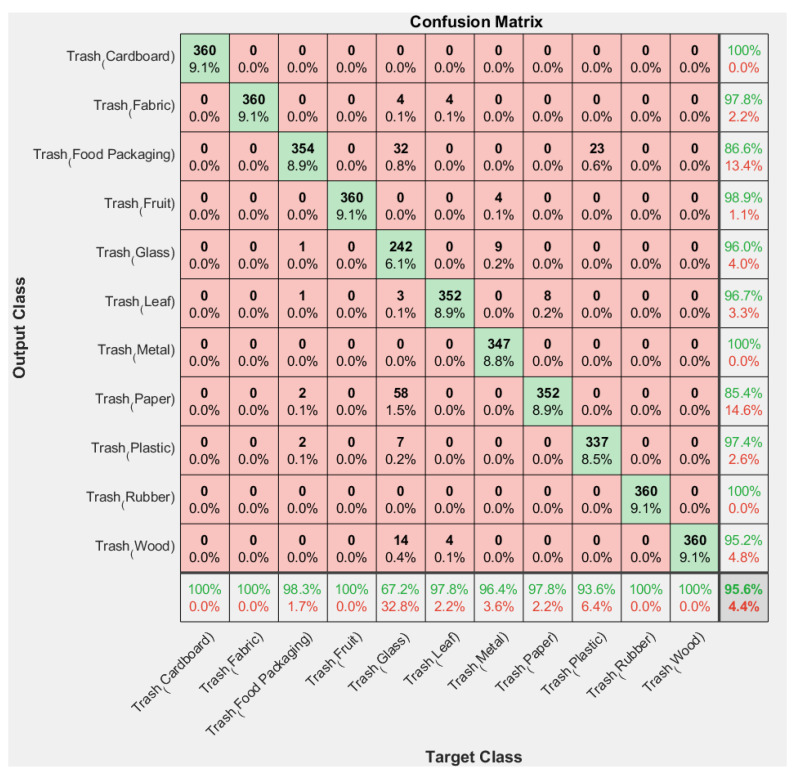
Result of confusion matrix trash using VGG16.

This trash object identification system is tested for its performance using a confusion matrix. The four pre-trained CNN architectures (AlexNet, VGG16, GoogleNet, and ResNet18) were tested according to their respective architectures. Figure 17 shows the results of the confusion matrix with VGG16.

Table 5 is the result of comparing the accuracy of the confusion matrix. The table shows that the AlexNet architecture has the lowest accuracy value, namely 80.5%, while the GoogleNet architecture has the highest value, namely 98.3%.

### 3.2. Trash Identification Test Using a Real-Time Camera

After the training process is complete, the identification test of the trash object is carried out. The test is carried out in real-time using a mobile device (iPhone) camera. The IP address used during testing is as follows.

**Figure 18:**
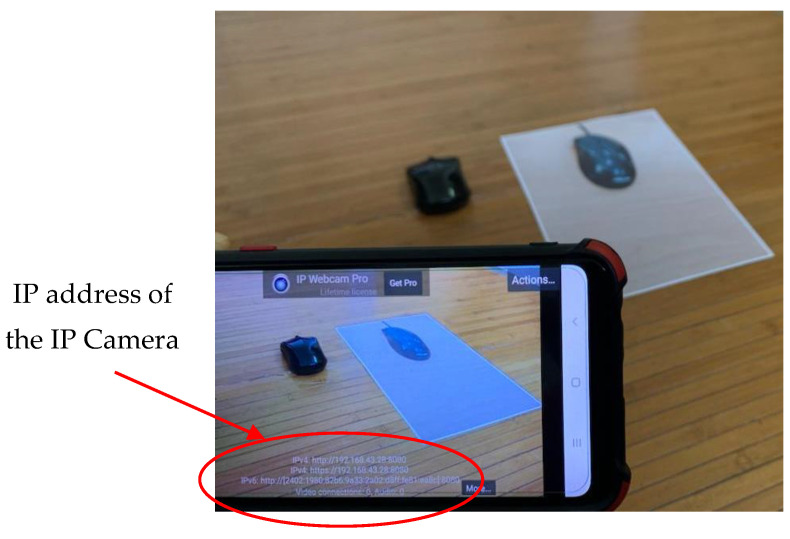
IP address of the IP camera displays on the mobile device (iPhone).

**Figure 19:**
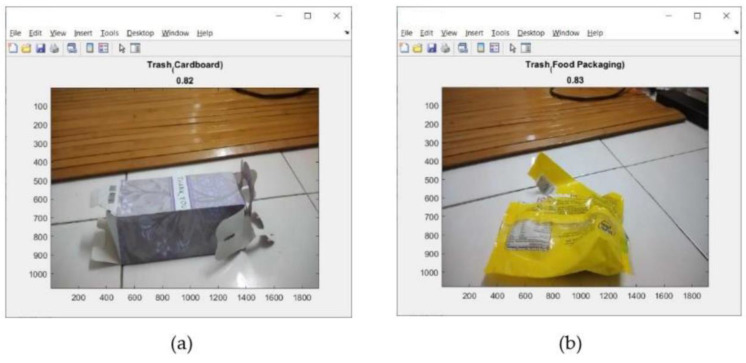
Results of trash identification using a real-time camera. (**a**) Cardboard; (**b**) food packaging.

**Figure 20:**
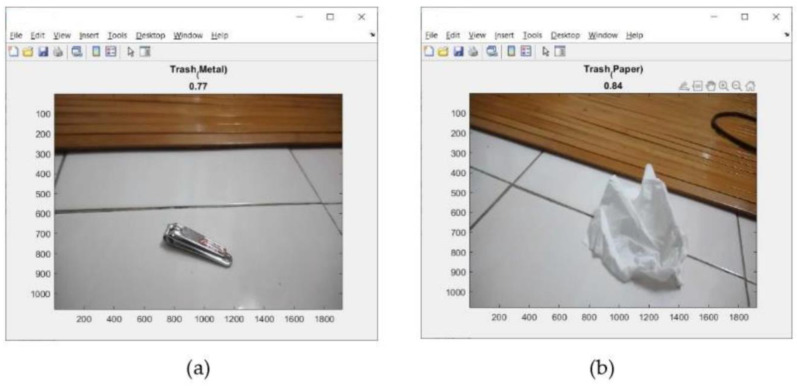
Results of trash identification using a real-time camera. (**a**) metal; (**b**) paper.

**Figure 21:**
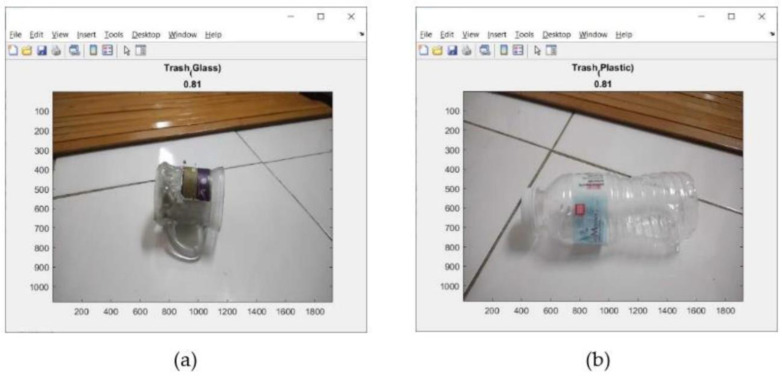
Results of trash identification using a real-time camera. (**a**) glass; (**b**) plastic.

Camera = ipcam (‘http://192.168.43.28:8080/video’ accessed on 18 October 2022); 

This address can be seen on the screen display of mobile device (iPhone) as shown in Figure 18. Tests of the 11 types of trash can be seen in Figure 19, Figure 20 and Figure 21. This test uses the AlexNet architecture.

**Figure 22:**
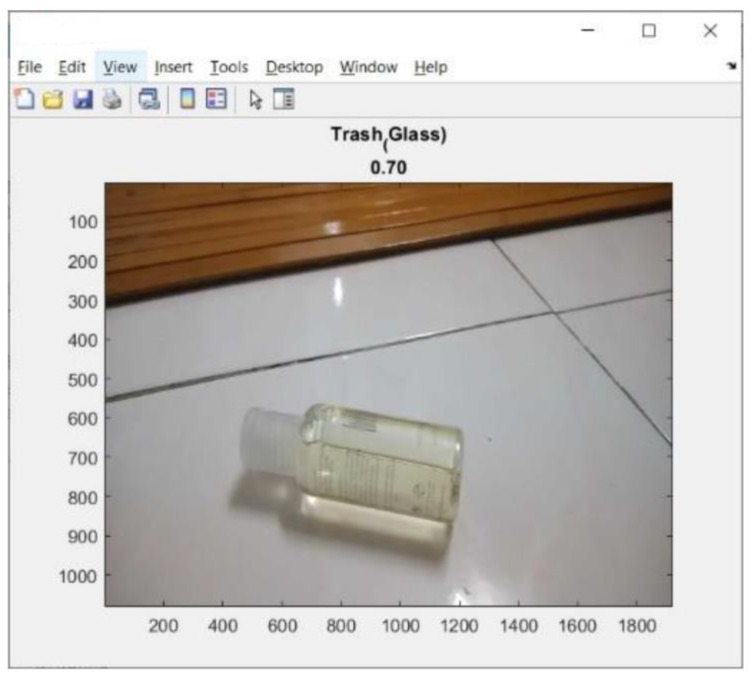
Result of the error trash identification.

At the time of testing, there was also an error in identifying the trash object, as shown in Figure 22. The trash object was identified as glass, even though the trash object was plastic.

The tests performed in Figure 19, Figure 20 and Figure 21 are used to test the performance of pre-trained CNN architectures (AlexNet, VGG16, GoogleNet, and ResNet18) in identifying trash in real time. The number tested was 411 pieces of trash. Then, record the total trash detected for each type of trash. This test used trash that had never been used in training. Table 6, Table 7, Table 8 and Table 9 show the results of the accuracy obtained by each pre-trained CNN architecture, while Table 10 shows the average accuracy that can be achieved by each pre-trained CNN architecture.

### 3.3. Result of a Fast Scanning Image

**Figure 23:**
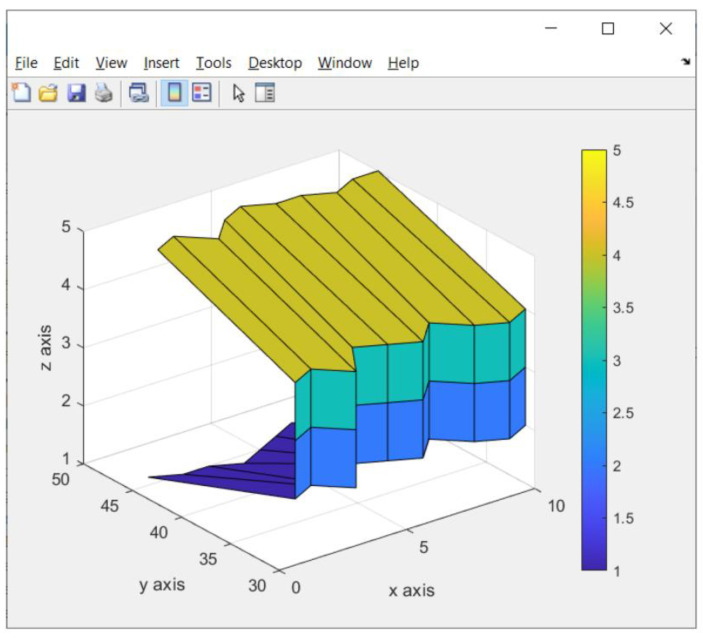
Result of fast scanning image of the trash object (box).

A fast scanning image is used to quickly ensure that the identified image is an image in 2D or 3D. The results of the fast scanning image can be seen in Figure 23.

In Figure 23, the results of the fast scanning image divide the value of the *z*-axis into 5. This value comes from the servo motor 2.

### 3.4. Result of Detail Scanning Image

After the fast scanning process states that an observed object is an object in 3D, the detailed scanning image process is carried out. The function of this detail scanning image is to ensure the robot gripper position when lifting trash.

**Figure 24:**
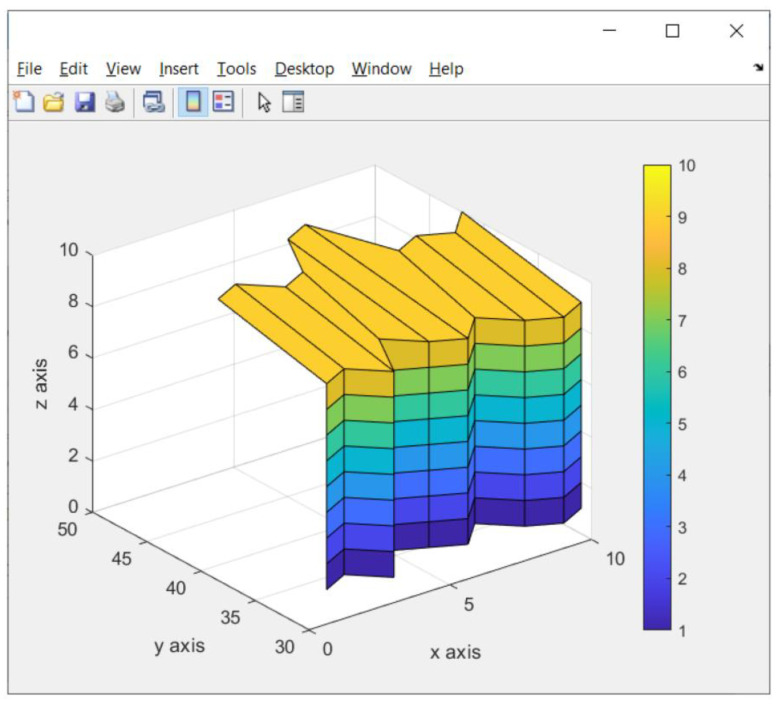
Result of detail scanning image of the trash object (box).

In Figure 24, it can be seen that the detailed scanning image results are better than the fast scanning image results. This is because the amount of data on the *y*-axis and *z*-axis are more than the amount of data on the *y*- and *z*-axis in fast scanning images.

### 3.5. Result of Time Speed Comparison between Fast Scanning Image and Detail Scanning Image

**Figure 25:**
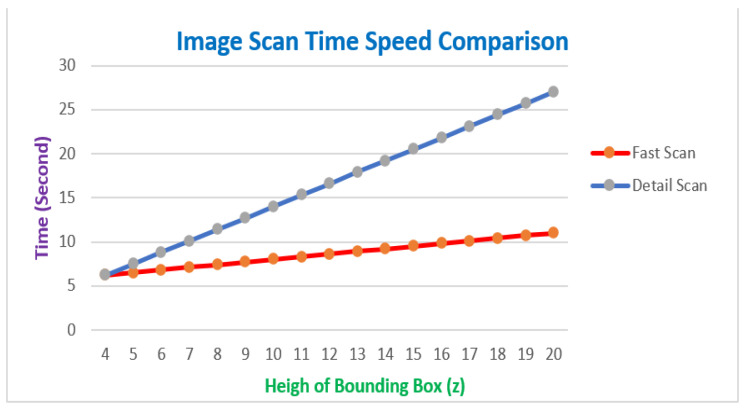
Result of fast scanning image and detail scanning image of the trash object.

One of the optimizations in the trash identification process is to make time efficient in identifying trash objects. Therefore, it is necessary to examine the difference between fast and detailed scanning images, as depicted in Figure 25. The efficiency of time consumption used in the trash identification process will be obtained from the results of this test. 

## 4. Discussion

In the initial experiment, the total images entered were 690 images with details: cardboard 50, fabric 15, food packaging 50, fruit 50, glass 50, leaf 165, metal 65, paper 100, plastic 120, rubber 15, and wood 15. In the results of training progress, accuracy ranges from 65.32% to 74.51%. Accuracy results on the confusion matrix ranged from 84.2% to 92.1%.

Furthermore, to increase the low accuracy value, the number of each data image in each class needs to be added, especially on images less than 20. Images of each class are added to a minimum of 150 images per class. Then, each image is augmented seven times so that each class has 1200 images. There is an increase in the training progress, with accuracy ranging from 77.54% to 86.38%. The accuracy in the confusion matrix increased from 80.5% to 98.3%. Thus, data images must be added to increase the accuracy of identifying the trash object. 

Based on Table 4 and Table 5, it can be seen that there is a linear correlation between the resulting accuracy values. The lowest accuracy value in Table 4 is also the lowest accuracy value in Table 5. Likewise, the highest accuracy value in Table 4 is also the highest accuracy value in Table 5. Thus, without testing the value of the confusion matrix, we can also predict the accuracy results on the confusion matrix by looking at the value of validation accuracy.

In the real-time trash identification test, there is a decrease in accuracy from each pre-trained CNN. The lowest accuracy is generated by AlexNet, which is equal to 79.410%, and the highest accuracy is generated by GoogleNet with accuracy of 96.513%. This is caused by the lighting factor when detecting the trash object. However, this accuracy value is already good for the identification process and can be continued for the next process, namely the 2D or 3D determination of the identified trash.

In Table 4, it can be seen that the accuracy of GoogleNet is higher than ResNet18, but ResNet18 has a much shorter training time compared to GoogleNet. This can also be taken into consideration in choosing the pre-trained CNN to be used. Moreover, if the system is made based on embedded systems.

Figure 11 illustrates the position of the LiDAR sensor during the scanning process. Based on the figure, there are three formulas used, namely:

1. If the object is straight in front of the LiDAR sensor, then the *y*-coordinate:

(6) y=distance value that measured by LiDAR sensor

2. If the object is on the front left side/the front right side of the LiDAR sensor, the *y*-coordinate value can be calculated by the formula:

(7) y=Cosθ×distance measured by LiDAR sensor

3. The formula can calculate the *x*-coordinate value:
  (8) x=Degree of servo Motor 1−90
  The value of 90 is due to servo motor 1 being set at 90 degrees. After all the *x* values are read and stored in the matrix variable, then the x value is added to the maximum *x* value with the formula:
  (9) x=x+maximum value of x

The formula aims to make the *x*-coordinate values all positive.

After the *x*-coordinate and *y*-coordinate values are obtained, then the *z*-value is obtained by the formula:

(10) z=Degree of servo Motor 2−90

After all the *z* values are read and stored in the matrix variable, then the *z* value is added to the maximum *z* value with the formula:

(11) z=z+maximum value of z

With a LiDAR accuracy of ±2 mm, the results of detail scanning images displayed using MATLAB are similar to the original object. However, because what is scanned is only the surface in front of the LiDAR sensor, the detailed scan only shows the object’s surface. At first, the data generated by the LiDAR sensor were still in the form of numbers containing the *y*-coordinate data after combining the data with the *x* coordinates (servo motor 1) and *z* coordinates (servo motor 2).

Scanning images in detail is relatively time-consuming because the frame rate capability of the LiDAR sensor is only 5 Hz. If every time it finds an object that is considered trash, the robot must scan a detailed image, so the decision to lift the object will take longer. For this reason, the system is equipped with fast scanning. With this fast scanning, if the object considered trash is not in 3D shape, then the lifting process by the robot gripper is ignored. This time efficiency can be performed, and the robot can search for the next trash object. 

In this study, the Sequential_Camera_LiDAR (SCL) method is proposed. This method is relatively simple, combining cameras and LiDAR sensors. A CNN processes the camera’s output, followed by a fast scanning process by the LiDAR sensor. A comparison of the proposed method with the previous method is in Table 1. However, this proposed study can be used in a house compound. The proposed method differs from the previous method in Table 1 (Early Fusion, Sequential Fusion, Late Fusion). These three methods are applied to vehicles that must have a relatively high speed compared to the speed of the robot. In addition, the vehicle’s response to obstacles in front of the camera is relatively faster than the robot’s movement in the house compound. So the proposed method is suitable for use in housing, but it is necessary to adjust the existing parameters for use on highways.

Indeed, in the real world, there is a possibility that 2D paper is also trash. However, the robot in this study has the disadvantage of not being able to lift 2D objects, such as 2D paper. It is due to the geometry of the robot gripper itself. It will be a gap for further research. In future studies, the robot gripper must be equipped with a suction system to lift 2D paper-shaped objects by being sucked on.

## 5. Conclusions

The optimization of the trash object detection system has been successfully carried out by using a fast scanning system based on bounding boxes. The time efficiency ranges from 13.33% to 59.26%, depending on the size of the detected object. The larger the object, the greater the time efficiency. The bigger the object, the more time it takes. Testing is limited to objects of 15 cm × 20 cm. To overcome this, in future research, we will try to use stereo LiDAR so that the scan time can be faster.

In testing the identification of trash objects using several CNN architectures—AlexNet, VGG16, GoogleNet, and ResNet18—all have a trash object identification accuracy of 80.5%, 95.6%, 98.3%, and 97.5%, respectively. This system uses a LiDAR sensor to ensure that the object is real or not real. The results of LiDAR scanning in graphic form can be adequately produced because the LiDAR sensor has a reading accuracy of 2 mm. The graph is the basis for determining the robot gripper position in lifting the trash object. Future research will focus on the trash collection process based on the resulting graph.

## Figures and Tables

**Table 1:** Comparison between the Sensor Fusion Method and the Proposed Study.

References	Sensors	Method	Comment
[23,24,25,26,27,28,29]	Camera RGB, LiDAR	Early Fusion	Data from RGB camera images and LiDAR sensors are directly input for deep learning and processed together. The result is complete depth.
[26,28,30]	Camera RGB, LiDAR	Sequential Fusion	The data from the RGB camera image are first processed by deep learning. The result is RGB depth. Then, these results and the LiDAR sensor data directly become the input for subsequent deep learning. Both are processed together, and the result is a complete depth.
[26,28,31]	Camera RGB, LiDAR	Late Fusion	The data from the RGB camera image are first processed by deep learning. The result is RGB depth. In addition, LiDAR sensor data are also processed by deep learning. The result is LiDAR depth. Furthermore, the respective outputs are processed together, resulting in complete depth.
Proposed	Camera RGB, LiDAR	Sequential_Camera_LiDAR (SCL): CNN + Fast Scanning Image + Detail Scanning Image	Image data from the RGB camera are processed using deep learning to detect the presence of trash objects. If what is detected is a trash object, then a Fast Scanning image is performed to ensure that the object is in 3D form. The scanning process uses a LiDAR sensor. If the object is 3D, then a detail scanning Image is performed to determine the correct position in lifting the trash object using a robot gripper.

**Table 2:** Training Parameter Used for Identification of Trash.

Parameter	AlexNet	VGG16	GoogleNet	Resnet18
MiniBatch size	5	5	5	5
Learning rate	0.0003	0.0003	0.0003	0.0003
Max epoch	6	6	6	6
Data augmented	Yes	Yes	Yes	Yes

**Table 3:** Main Parameters of TF40.

Parameter Name		Standard Version
Product performance	Range	0.04–40 m 90% reflectivity, 0.04–20 m 10% reflectivity
	Accuracy	±2 mm
	Distance resolution	1 mm
	Frame rate	5 Hz
Optical parameters	Light source	LD
	Wavelength	635 nm
	Laser class	CLASS 2 (EN 60825)
	Detection angle	<1 mrad
Electrical parameters	Supply voltage	3.3 V
	Average current	≤180 mA
	Power consumption	≤0.6 W
	Communication voltage level	LVTTL (3.3 V)

**Table 4:** Training Progress Result.

CNN Architecture	Training Time (s)	Validation Accuracy (%)
AlexNet	229	77.54
VGG16	894	79.09
GoogleNet	581	86.38
ResNet18	335	80.75

**Table 5:** Comparison of the Confusion Matrix.

CNN Architecture	Accuracy (%)
AlexNet	80.5
VGG16	95.6
GoogleNet	98.3
ResNet18	97.5

**Table 6:** The Accuracy of 11 Types of Trash Using a Real-Time Camera with Pre-Trained AlexNet.

Types of Trash	Number of Real Trash Tests	Total Trash is Correctly Detected	Accuracy (%)
Cardboard	45	37	82.22222222
Fabric	17	13	76.47058824
Food Packaging	63	52	82.53968254
Fruit	21	17	80.95238095
Glass	34	27	79.41176471
Leaf	56	46	82.14285714
Metal	21	16	76.19047619
Paper	61	51	83.60655738
Plastic	52	42	80.76923077
Rubber	14	10	71.42857143
Wood	27	21	77.77777778
Overall	411	332	79.41019176

**Table 7:** The Accuracy of 11 Types of Trash Using a Real-Time Camera with Pre-Trained VGG16.

Types of Trash	Number of Real Trash Tests	Total Trash is Correctly Detected	Accuracy (%)
Cardboard	45	40	88.88888889
Fabric	17	14	82.35294118
Food Packaging	63	57	90.47619048
Fruit	21	19	90.47619048
Glass	34	30	88.23529412
Leaf	56	49	87.50000000
Metal	21	18	85.71428571
Paper	61	54	88.52459016
Plastic	52	45	86.53846154
Rubber	14	11	78.57142857
Wood	27	23	85.18518519
Overall	411	360	86.58758694

**Table 8:** The Accuracy of 11 Types of Trash Using a Real-Time Camera with Pre-Trained GoogleNet.

Types of Trash	Number of Real Trash Tests	Total Trash is Correctly Detected	Accuracy (%)
Cardboard	45	44	97.77777778
Fabric	17	16	94.11764706
Food Packaging	63	62	98.41269841
Fruit	21	20	95.23809524
Glass	34	33	97.05882353
Leaf	56	55	98.21428571
Metal	21	20	95.23809524
Paper	61	60	98.36065574
Plastic	52	51	98.07692308
Rubber	14	13	92.85714286
Wood	27	26	96.29629630
Overall	411	400	96.51349463

**Table 9:** The Accuracy of 11 Types of Trash Using a Real-Time Camera with Pre-Trained ResNet18.

Types of Trash	Number of Real Trash Tests	Total Trash is Correctly Detected	Accuracy (%)
Cardboard	45	43	95.55555556
Fabric	17	15	88.23529412
Food Packaging	63	61	96.82539683
Fruit	21	20	95.23809524
Glass	34	33	97.05882353
Leaf	56	54	96.42857143
Metal	21	20	95.23809524
Paper	61	59	96.72131148
Plastic	52	50	96.15384615
Rubber	14	13	92.85714286
Wood	27	26	96.2962963
Overall	411	394	95.14622079

**Table 10:** The Average Accuracy of Pre-Trained CNN in Real-Time Trash Identification.

CNN Architecture	Average Accuracy (%)
AlexNet	79.410
VGG16	86.588
GoogleNet	96.513
ResNet18	95.146

## Data Availability

The data that support the findings of this study are available from the corresponding author upon reasonable request.
